# Patient and clinician perceptions, expectations, and usability of ankle exoskeletons for daily living: a mixed-methods survey study

**DOI:** 10.3389/fdgth.2026.1745605

**Published:** 2026-05-14

**Authors:** Daniel Gomez-Vargas, Vanessa Uchenna, Manon Bocahut, Flavio Roberti, Ricardo Carelli, Marcela Munera, Carlos A. Cifuentes

**Affiliations:** 1 Institute of Automatics, National University of San Juan, San Juan, Argentina; 2 Bristol Robotics Laboratory, University of the West of England, Bristol, United Kingdom; 3 Graduate School of Engineering, EPF–Engineering School (EPF), Cachan, France

**Keywords:** activities of daily life (ADL), ankle exoskeleton, KAP, perception, survey, SUS, usability

## Abstract

Ankle exoskeletons offer promising support for individuals with chronic foot drop, yet user and clinician perspectives on their use in daily living remain underexplored. Related studies on lower limb exoskeletons have typically assessed user perceptions following direct physical interaction with devices, which may influence feedback and its interpretation. In contrast, this study aimed to assess perceptions, expectations, and usability through a convergent mixed-methods approach involving 43 participants (i.e., 27 patients diagnosed with foot drop and 16 clinicians), without requiring hands-on experience. Participants completed a pre-exposure Knowledge, Attitudes, and Practices (KAP) survey, viewed educational videos on ankle exoskeleton technology, and subsequently completed a follow-up survey assessing expectations, potential daily applications, and usability using the System Usability Scale (SUS). Quantitative analysis revealed that 70.4% of patients were initially unaware of exoskeletons, and 81.5% reported no understanding of how they function. Clinicians demonstrated significantly higher knowledge scores than patients (U=60, p<0.001, r=0.62), while no statistically significant difference was found in attitude-related measures between groups (U=264, p=0.18, r=0.20). Both groups reported generally positive perceptions of exoskeletons for improving mobility and independence. However, concerns regarding comfort, aesthetics, potential skin irritation, and integration into daily activities were identified as relevant barriers. Additionally, practical challenges such as cost, device bulkiness, and usability were noted by both groups. Qualitative findings complemented these results by providing further insight into user concerns and design expectations. These findings highlight key factors influencing the potential adoption of ankle exoskeletons and underscore the importance of user-centred design, extended trial opportunities, and adaptable, practical solutions for both clinical and home-based applications.

## 1 Introduction

Foot drop is a disorder characterised by the inability to raise the forefoot effectively (1, 2). This condition is frequently the result of neurological pathologies such as stroke, multiple sclerosis, or spinal cord injuries (3, 4). Foot drop significantly impairs gait, increases the risk of falls, and restricts patients’ independence (5, 6). Traditional management of chronic foot drop has been addressed mainly through (1) ankle-foot orthoses (AFOs), (2) pharmacological or surgical treatments, and (3) physical therapy (7).

AFOs, predominantly fabricated from plastic or carbon fibre, keep the ankle in a neutral position, thus maintaining clearance between forefoot and ground during swing phase (8). These orthoses improve gait stability but have certain limitations related to the inability to assist the ankle motions, causing an abnormal gait pattern (8). Pharmacological treatments are focused on reducing spasticity, although they do not directly address the mechanical deficits of foot drop (9). Surgical interventions, including tendon transfers or nerve decompression, can restore some function but carry risks such as infection, nerve damage, and variable outcomes depending on the patient’s condition (10). Physical therapy aims to strengthen the dorsiflexor muscles and improve gait through exercises and neuromuscular training. However, its effectiveness is often limited in severe or chronic foot drop. Additionally, patient adherence to long-term therapy can be challenging, potentially reducing the efficacy of this approach (11).

The limitations of traditional treatments have led to a growing interest in ankle exoskeletons. These devices have been designed to compensate for muscle weakness and support the impaired ankle functions, improving individual mobility and facilitating the proper execution of other activities of daily living (ADLs) (12, 13). Multiple studies have reported promising outcomes of ankle exoskeleton’s use in terms of motor recovery and independence (14, 15). However, their approval by end users, i.e., patients and healthcare professionals, is critical for their successful adoption and integration in clinical and daily settings (16). Yet, despite their importance, studies focused explicitly on user acceptability remain scarce (16). In this context, user perception plays a pivotal role in determining the acceptance of an ankle exoskeleton, influencing both the user’s motivation to engage with the device and the long-term sustainability of its use (17). This concept also encompasses characteristics such as initial expectations, psychological acceptability, obstacles, and apprehensions related to daily use (17, 18).

To the best of the authors’ knowledge, no studies to date have specifically examined ankle exoskeletons in this context. Therefore, the studies presented below and in the subsequent section pertain more broadly to lower limb exoskeletons. In this light, current research highlights a significant diversity in assessing the perception of exoskeletons. Moreover, the timing of user perception assessment reported in the literature significantly affects the nature of the findings and their interpretation, thus requiring careful consideration. Most studies inform exoskeleton perception based on user feedback after a direct interaction or device demonstration (5, 6, 19–21). Some studies aim to assess user perception before device usage (17, 18, 22, 23), while others, such as (24), assess for both cases (i.e., before and after). Nonetheless, participants were typically first exposed to information about the technology, which may have influenced their initial responses. This way, existing studies could provide a user perception of a specific exoskeleton device, rather than addressing the broader concept of wearable robotic assistance. As a result, they could offer limited insight into whether users and clinicians would be willing to adopt this technology for routine, daily use. Moreover, most current research is conducted in clinical environments, where the evaluation is often confined to short-term, controlled settings. Consequently, the applicability of these findings to activities of daily living is not directly assessed and is extrapolated without considering the unique demands of real-world use in some cases.

To address these identified gaps, this study aimed to assess the perceptions, expectations, and usability considerations of clinicians and patients regarding the ankle exoskeletons’ use in daily living activities, using a two-part mixed-methods survey. The first survey, designed as a Knowledge, Attitudes, and Practices (KAP) assessment, aimed to capture baseline insights into participants’ familiarity, prior experience, and preferences concerning ankle exoskeletons. Following this, participants watched informative videos outlining the concept of lower limb and ankle exoskeletons, their functionalities, various types, real-life application examples, and summarised evidence on their use. A second survey was carried out to evaluate participants’ updated views, including desirable features, potential applications in ADLs, and overall receptiveness. Finally, this study included a System Usability Scale (SUS)-based questionnaire tailored to ankle exoskeletons to gauge perceived usability. Thus, this work aims to provide an understanding of the factors influencing the acceptance and potential integration of ankle exoskeletons into clinical and personal routine use.

## 2 Related works

As mentioned previously, no studies to date have specifically investigated user perceptions of ankle exoskeletons. However, several relevant works reported in the literature have examined this topic in the broader context of lower limb exoskeletons. These studies have involved different methodological tools, including questionnaires, semi-structured interviews, and focus groups. Notably, several works that collected quantitative data also incorporated in-depth qualitative analyses (18–20), which were often complemented by descriptive statistical techniques (5, 6).

Reicherzer et al. (20) conducted interviews to assess perceptions and concerns regarding the use of the Myosuit exoskeleton, applying a qualitative thematic analysis based on the Theoretical Domains framework, but did not incorporate any statistical analysis. They found that users initially had limited familiarity and mixed perspectives, while healthcare professionals reported barriers related to technical complexity or costs. A study by Mitchell et al. (19) employed multidisciplinary focus groups to assess perceptions of rehabilitation technologies (e.g., exoskeletons, brain-computer interfaces, virtual reality) applied to individuals with spinal cord injury. The analysis focused solely on subjective themes, such as overall cost, perceived benefits, usability, and accessibility of these technologies.

Shore et al. (18) conducted a qualitative analysis using grounded theory, in-depth interviews, and thematic coding to evaluate initial perceptions and adaptation needs related to assistive technologies, including exoskeletons. This study revealed a significant initial lack of awareness and the need for clear explanations to promote acceptance. Similarly, Herrera-Valenzuela et al. (22) employed a qualitative analysis through direct interviews with clinicians and patients who suffered spinal cord injuries, highlighting the importance of actively involving experienced users in the lower limb exoskeletons’ design.

On the other hand, (6) conducted a mixed approach using semi-structured interviews and the 10-point Likert scale to assess the gait’s comfort and naturalness after direct exposure to the EKSO exoskeleton in stroke survivors. This study included a descriptive statistical analysis supplemented by a Spearman correlation test, highlighting the significant relationship between the disability level and the positive perception concerning the exoskeleton’s assistance.

There is considerable variability in the populations examined across previous studies. For instance, Shore et al. (18) and Reicherzer et al. (20) analysed the perceptions of independent older adults. McDonald et al. (6) considered people with neurological conditions such as stroke, and works by Camardella et al. (5), Herrera-Valenzuela et al. (22) and Mitchell et al. (19) involved healthy participants, clinicians and multidisciplinary experts, respectively.

The literature emphasises the importance of initial familiarisation with the technology and awareness of its potential benefits in enhancing acceptance and adoption. Furthermore, several barriers have been identified as limiting factors, including the complexity of use, costs, or even psychological aspects (19, 20). On the other hand, the principal limitations identified in these studies relate to several key aspects. First, the surveyed populations are often highly specific, e.g., studies focusing exclusively on older adults. Thus, it restricts the generalisability of the findings to broader groups. Second, most works assess user perceptions at a single time point, i.e., before or after exposure to information or device use. Thereby, this limits the ability to examine how perceptions evolve over time. Lastly, the absence of baseline evaluations before information exposure hinders a clear understanding of the role that awareness and prior knowledge may play in shaping attitudes towards the technology.

## 3 Methodology

### 3.1 Research design

This study follows a convergent mixed-methods design, in which quantitative and qualitative data were collected via surveys online within the same instruments and analysed in parallel. The qualitative component was used to complement the quantitative findings, providing additional insight into participants’ perceptions, expectations, and concerns related to ankle exoskeleton technology. The research design included three main components: an initial online questionnaire, a video session introducing ankle exoskeleton technology, and a follow-up assessment survey. The first survey incorporated a Knowledge, Attitudes, and Practices (KAP) questionnaire to capture baseline familiarity, beliefs, and preferences regarding ankle exoskeletons. Following the video session, which presented key functionalities, types, user examples, and general outcomes of exoskeleton use, participants completed a second survey to assess their updated perceptions, expectations, and preferences for potential applications in daily living. Additionally, a System Usability Scale (SUS) questionnaire measured the perceived usability of the technology.

Integration of quantitative and qualitative findings was conducted at the interpretation stage. Qualitative responses were used to complement and contextualise quantitative results by providing explanatory insights into participants’ perspectives. Convergences between data types were identified to strengthen key findings, while qualitative data also helped to clarify underlying reasons behind observed trends in the quantitative analysis.

The survey instruments were developed for the purposes of this study by adapting established approaches. However, no formal pilot testing or psychometric validation was conducted prior to deployment. Therefore, the measures should be interpreted within the exploratory scope of the study.

### 3.2 Participants

This study involved a total of 43 participants, including 27 individuals diagnosed with foot drop and 16 clinicians, such as physiotherapists and rehabilitation professionals (see Table 1). In the UK context, the primary qualification required to practise medicine (e.g., MBBS or MBChB) is obtained through an undergraduate-entry medical programme. Therefore, clinicians indicating an “undergraduate” education level may hold a professional medical degree. The inclusion of both end-users and healthcare providers reflects the study’s aim to engage the primary stakeholders in ankle exoskeleton adoption, incorporating diverse perspectives and needs. All participants were recruited based on defined eligibility criteria, which are detailed below.

**Table 1:** Demographic and clinical characteristics of study participants. For patients with foot drop, data include time since diagnosis, current treatments, and use of walking support. For clinicians, the table summarises professional roles and years of experience.

Foot-drop patients			Clinicians		
	Number	%		Number	%
Gender			Gender		
Male	2	7	Male	10	63
Female	25	93	Female	6	38
Age (years)			Age (years)		
18–30	2	7	18–30	5	31
31–40	1	4	31–40	5	31
41–50	4	15	41–50	6	38
51–60	6	22	Professional role		
61–70	10	37	Physical therapist	5	31
71+	4	15	Nurse	3	19
Diagnosis (years)			Physician	10	63
<1	8	30	Education level		
1–5	10	37	Undergraduate	12	75
>5	9	33	Postgraduate	4	25
Treatments			Practice setting		
Physical therapy	26	96	Private	10	63
Orthotics	20	74	Public	6	38
Surgery	7	26	Experience (years)		
Others	8	30	2–5	11	69
* Technological	2	7	6–10	2	13
* Pharmacological	2	7	11+	3	19
* Exercise-based	4	15	Mobility cases (%)		
Walking support			<15	10	63
Orthosis or brace	25	93	15–30	3	19
Gait Aid	12	44	>30	3	19

#### 3.2.1 Foot drop patients

1. Inclusion criteria
  (a) Diagnosed with a neurological condition resulting in foot drop (e.g., nerve/ muscle damage, stroke, spinal cord injury, multiple sclerosis, cerebral palsy).
  (b) Aged 18 years or older.
  (c) Self-reported sufficient cognitive and communication abilities to understand and respond to study questionnaires.
2. Exclusion criteria
  (a) Severe cognitive or communication impairments (including insufficient language proficiency) that would prevent understanding study materials, the video content, questionnaires, or providing informed consent.
  (b) Unstable medical conditions or comorbidities (e.g., acute psychiatric crisis, severe pain flare, symptomatic uncontrolled hypertension, acute musculoskeletal injury) that would contraindicate participation or potentially confound study results.
  Severe visual or auditory impairments that would prevent viewing video simulations or completing questionnaires.

#### 3.2.2 Clinicians

1. Inclusion criteria
  (a) Licensed physical therapist, occupational therapist, physiatrist, neurosurgeon, orthopaedic surgeon, physiotherapy nurse, or physician.
  (b) Minimum of 2 years of experience working with patients with neurological conditions causing foot drop (e.g., stroke, spinal cord injury, multiple sclerosis, cerebral palsy).
2. Exclusion criteria
  (a) Non-clinical staff or students without direct patient care experience.
  (b) Clinicians who primarily work with patient populations unrelated to neurological conditions causing foot drop.

### 3.3 Recruitment procedure

Participants were recruited through a combination of targeted online outreach and professional collaboration. The recruitment process aimed to ensure representation from two key stakeholder groups: individuals with foot drop and clinicians involved in neurological rehabilitation. Initial outreach efforts were conducted via social media platforms, where several relevant community and professional groups were identified. These included physiotherapy networks, groups for nurses and orthopaedic professionals, and UK-based foot drop support communities. A message was posted across these platforms, briefly describing the study and inviting interested individuals to participate. Individuals who expressed interest were subsequently contacted directly and guided through the data collection procedure outlined in the following section.

### 3.4 Data collection procedure

The data collection process was conducted entirely online to ensure accessibility and convenience for participants, allowing them to complete the study components remotely and at their own pace. This procedure consisted of five sequential steps:

**Invitation and informed consent:** Interested participants were initially contacted via email, which included a brief overview of the study objectives, procedures, inclusion and exclusion criteria. The email also contained a hyperlink directing them to an online informed consent form. Participants were required to review and electronically agree to the consent terms before proceeding to the next phase of the study.
**Pre-simulation survey:** Upon providing consent, participants completed a pre-simulation questionnaire (KAP survey) designed to collect baseline data. This survey gathered demographic information, data on participants’ experience with ADLs, and their familiarity and engagement with assistive technologies. The questionnaire combined multiple-choice items, Likert-scale questions, and open-ended responses, allowing for quantitative and qualitative insights.
**Video simulation exposure:** After completing the pre-survey, participants were exposed to multiple short online videos showcasing lower-limb and ankle exoskeleton technologies used in rehabilitation and assistance. The selected videos included outcomes from the commercial ReWalk exoskeleton (https://www.youtube.com/watch?v=DyAJZQPHk6c), the ReWalk ankle exosuit (https://www.youtube.com/watch?v=ZmCSTJQAcCE), the Ankle Ti-Rex exoskeleton developed by the University of Illinois Chicago (https://www.youtube.com/watch?v=-FS-x_YpkOI), and an ankle exoskeleton developed by the Colombian School of Engineering (https://www.youtube.com/watch?v=h_nAn3HbQ2o). The selection intentionally included both commercially available systems and research-stage prototypes in order to present a broader representation of current technological developments in ankle exoskeletons. This approach allowed participants to reflect not only on existing commercial solutions but also on emerging research technologies, with the aim of identifying user perceptions, concerns, and design considerations that could inform future development and facilitate earlier adoption of these assistive technologies. The videos emphasised device functionalities, potential benefits, and real-life applications to improve mobility, with the intent of standardising participants’ understanding and stimulating informed reflection.
**Post-simulation survey:** Following the video session, participants were directed to a second survey aimed at assessing their updated perceptions and expectations regarding the ankle exoskeleton’s use. This phase also included a SUS survey, allowing participants to reflect on their anticipated experience with the device. These surveys explored preferred characteristics of the technology, potential applications in daily living, and overall attitudes towards adoption. As in the pre-survey, the questionnaires included a combination of multiple-choice, Likert-scale, and open-ended items.
**Submission:** Upon completing the post-simulation survey, participants submitted their responses electronically via the Qualtrics XM online platform.

All participants completed both the KAP questionnaire and the post-simulation survey following the video session. Only fully completed responses (see Table 1) were included in the analysis presented in the following sections. Responses were collected sequentially from the same individuals.

### 3.5 Ethics statement

This protocol was approved by the University of the West of England Ethics Committee. All participant interactions and data collection adhered to established ethical guidelines, ensuring informed, voluntary, and confidential participation.

### 3.6 Data analysis

The analysis of collected data involved both quantitative and qualitative methods to comprehensively evaluate participant responses across demographics, perception measures, and usability evaluations. The primary inferential objective of this study was to examine differences between participant groups (patients and clinicians). Although data were collected sequentially from the same individuals, the pre- and post-surveys were designed to assess different constructs (i.e., baseline knowledge and attitudes vs. post-exposure perceptions, expectations, and usability). Therefore, responses were analysed as complementary datasets rather than paired within-subject measures, and no formal statistical comparison of pre–post changes was performed. The results are thus interpreted as descriptive insights at each stage rather than longitudinal effects.

#### 3.6.1 Descriptive statistics

This analysis incorporated the descriptive statistical findings derived from two data sources: the pre-simulation through of the KAP survey and the post-simulation using the Reactions to Video Simulation survey. These instruments were designed to capture both baseline perceptions and changes in participant views after being exposed to a simulated experience of ankle exoskeleton use. To facilitate this comparison, numerical values were assigned to both survey responses, allowing statistical analysis of trends between patients and clinicians.

Although the KAP surveys for patients and clinicians were tailored to their respective roles, this study analysed the data collectively to explore shared themes and contrasts in knowledge, attitudes, and practices related to ankle exoskeletons. The presented outcomes aligned key variables from both surveys to enable comparative interpretation and highlight parallel insights and differences between the two groups. In the knowledge domain specifically, a composite score was calculated for each patient by summing three items: (1) awareness of exoskeletons in rehabilitation, (2) understanding of how they work, and (3) familiarity with their use to support walking. Each item was scored on an ordinal scale reflecting increasing knowledge. To evaluate the internal consistency of this composite measure, Cronbach’s alpha and McDonald’s omega were computed. Cronbach’s alpha provides an estimate of reliability under the assumption of equal item contributions (tau-equivalence), whereas McDonald’s omega offers a more robust reliability estimate that does not rely on this assumption and accounts for the underlying factor structure. By contrast, clinicians answered a single-item Likert-scale question on familiarity, rescaled to match the range of the patient composite scores.For the attitudes domain, patients rated their willingness to use an exoskeleton if it could improve mobility, while clinicians indicated their agreement with the statement that exoskeletons can improve patients’ mobility. Responses for both groups were scored on comparable 5-point ordinal scales.

In statistical terms, the selection of tests was based on the nature of the data and sample size considerations. Several variables were derived from ordinal Likert-type scales or composite scores based on ordinal items, which do not strictly satisfy the assumptions of interval-level measurement required for parametric tests. In addition, distributional characteristics were assessed using the Shapiro–Wilk test to evaluate normality. Based on these considerations, non-parametric tests (e.g., Mann–Whitney *U* test) were used for group comparisons. Moreover, to summarise central tendency and variability within each group, median values and interquartile ranges (IQR) were reported in the results section.

#### 3.6.2 Thematic analysis

Responses to open-ended questions from both the pre- and post-simulation surveys were analysed using an inductive thematic analysis approach following the framework proposed by Braun and Clarke (25). This method was selected due to its flexibility and suitability for exploratory research aimed at identifying patterns in participants’ perceptions, expectations, and concerns.

The analysis was conducted through a structured but flexible process. First, all responses were reviewed to achieve familiarisation with the data. Initial codes were then generated inductively from the data without the use of predefined categories. These codes were subsequently grouped into broader themes reflecting recurring patterns across participant responses. A consensus-based approach was adopted during coding and theme development. Codes and themes were discussed and refined collaboratively among the research team to ensure consistency and interpretative coherence. No formal intercoder reliability metrics were computed, in line with the reflexive thematic analysis approach, which emphasises researcher interpretation and reflexivity over statistical agreement.

The coding and organisation of responses were performed manually using spreadsheet software (Microsoft Excel), enabling systematic categorisation and tracking of themes across participants and timepoints. Examples of representative responses, initial codes, and resulting themes are provided in the Supplementary Material to illustrate the coding and theme development process.

#### 3.6.3 System usability scale (SUS)

SUS scores were calculated to assess the perceived usability of ankle exoskeletons following the visual stimulation. The distribution of SUS scores was assessed for normality using the Shapiro–Wilk test. As the test reported normally distributed scores, an independent samples *t*-test was performed to compare mean usability perceptions between clinicians and patients, enabling statistical assessment of group-level differences.

## 4 Results

### 4.1 Descriptive statistics

#### 4.1.1 Pre-simulation

It is important to note that the constructs assessed for patients and clinicians in this section are related but not conceptually equivalent. Specifically, the patient measure reflects behavioural intention (i.e., willingness to use an exoskeleton), whereas the clinician measure reflects efficacy beliefs regarding the technology (i.e., perceived impact on patient outcomes). While both constructs relate to attitudes toward ankle exoskeletons, they capture different perspectives from each stakeholder group. Therefore, comparisons between patients and clinicians should be interpreted as complementary insights rather than direct equivalence of the same underlying construct.

**Figure 1:**
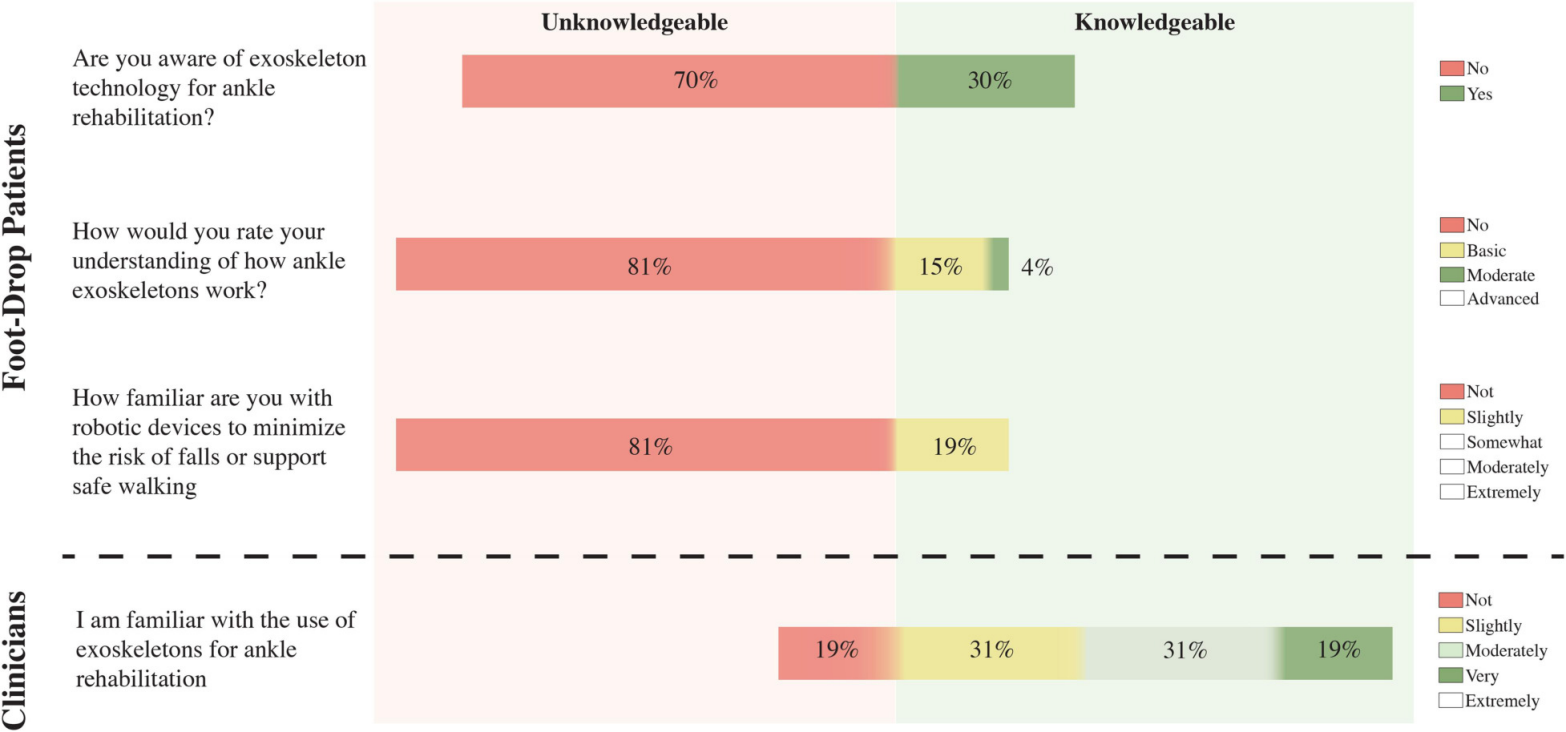
Knowledge of patients and clinicians regarding ankle exoskeleton use. The left side presents the questions used to assess each group’s knowledge. White boxes indicate unselected response options.

Following the KAP survey, Figure 1(top) shows the participants’ knowledge regarding the lower limb and ankle exoskeleton’s concept. This study found that 70% of patients were unaware of exoskeletons. Among those who were aware, the majority (*n* = 7) had obtained information from the internet, while one patient reported personal experience with the technology. Regarding the understanding of exoskeletons, 81% of patients reported no understanding of exoskeletons, with a similar percentage indicating no familiarity with the technology. Similarly, Figure 1(bottom) also shows that 19% of clinicians reported no familiarity with exoskeleton technology. In contrast, the majority indicated being either somewhat familiar (31%) or moderately familiar (31%), while 19% described themselves as extremely familiar.

The internal consistency of the patient knowledge composite score was acceptable, with Cronbach’s α=0.76 and McDonald’s $\omega_{t}=0.84$. The omega hierarchical coefficient was ($\omega_{h}=0.09$), suggesting limited variance attributable to a general factor. Concerning the normality assessment, the Shapiro-Wilk test indicated that patient knowledge scores significantly deviated from normality (W=0.77, p<0.001), whereas clinician scores did not show significant deviation (W=0.89, p=0.060). Therefore, knowledge scores were compared between patients (Median = 0, IQR = 0–1) and clinicians (Median = 6, IQR = 4–8) using the Mann-Whitney *U* test, revealing a statistically significant difference between the two groups (U=60, p<0.001, r=0.62), with higher scores observed in clinicians. These findings suggest a significant gap in familiarity with exoskeleton use in rehabilitation, favouring the clinician group. It should be noted that the comparison between patient and clinician knowledge measures is approximate, as it involves a multi-item composite for patients and a single-item measure for clinicians. Therefore, these results should be interpreted as indicative rather than strictly comparable constructs.

**Figure 2:**
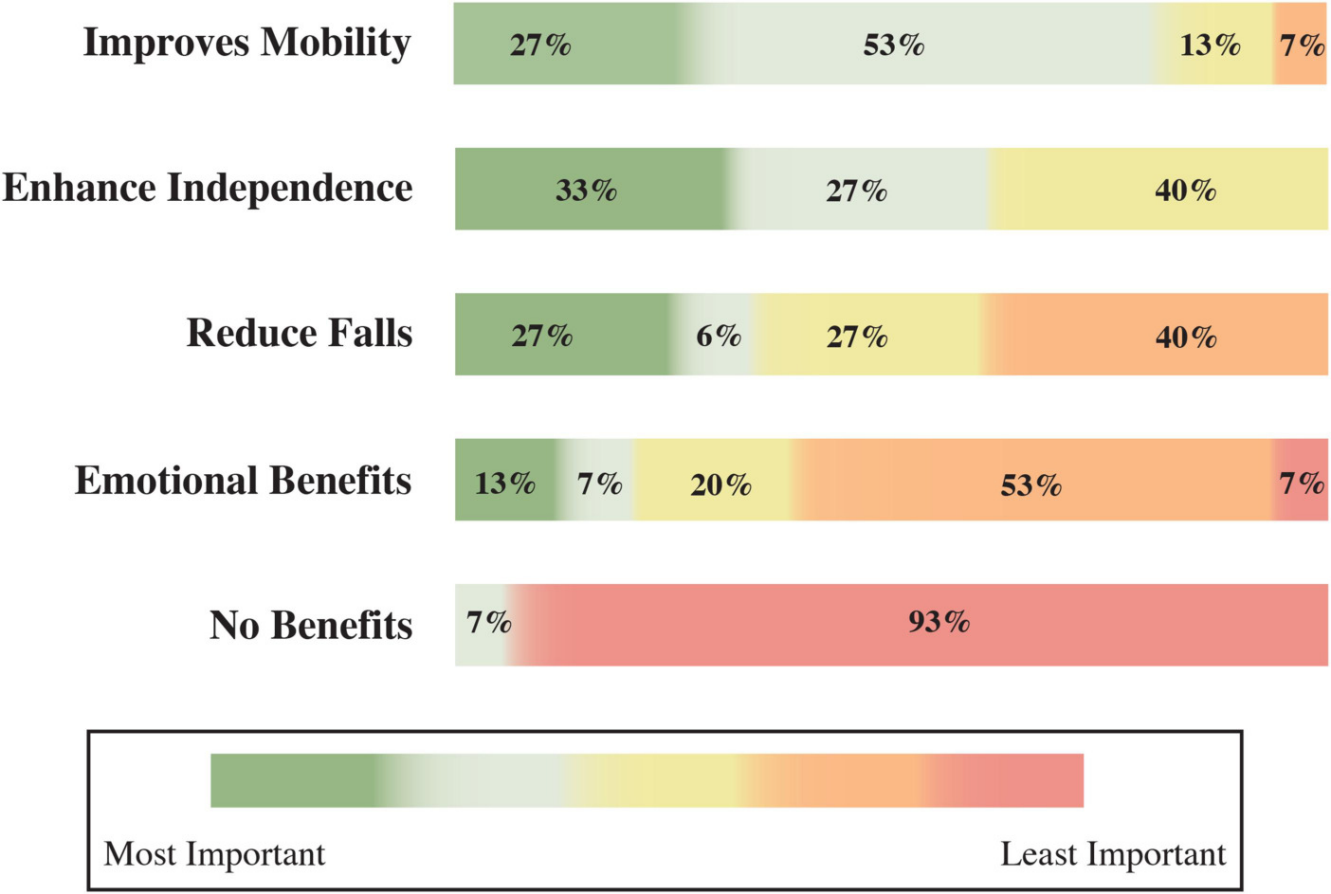
Ranking of clinicians’ perceived benefit of using an exoskeleton from least important to most important.

Consistent with participants’ reported knowledge, Figure 2 presents clinicians’ perceived benefits of using an exoskeleton, ranked from most to least important. Improving mobility emerges as the top benefit, with 80% ranking it highly important. Enhancing independence follows closely, with 60% viewing it as crucial. Reducing falls registers a benefit in 54% of respondents, and emotional benefits are less prioritised, with only 20%.

**Figure 3:**
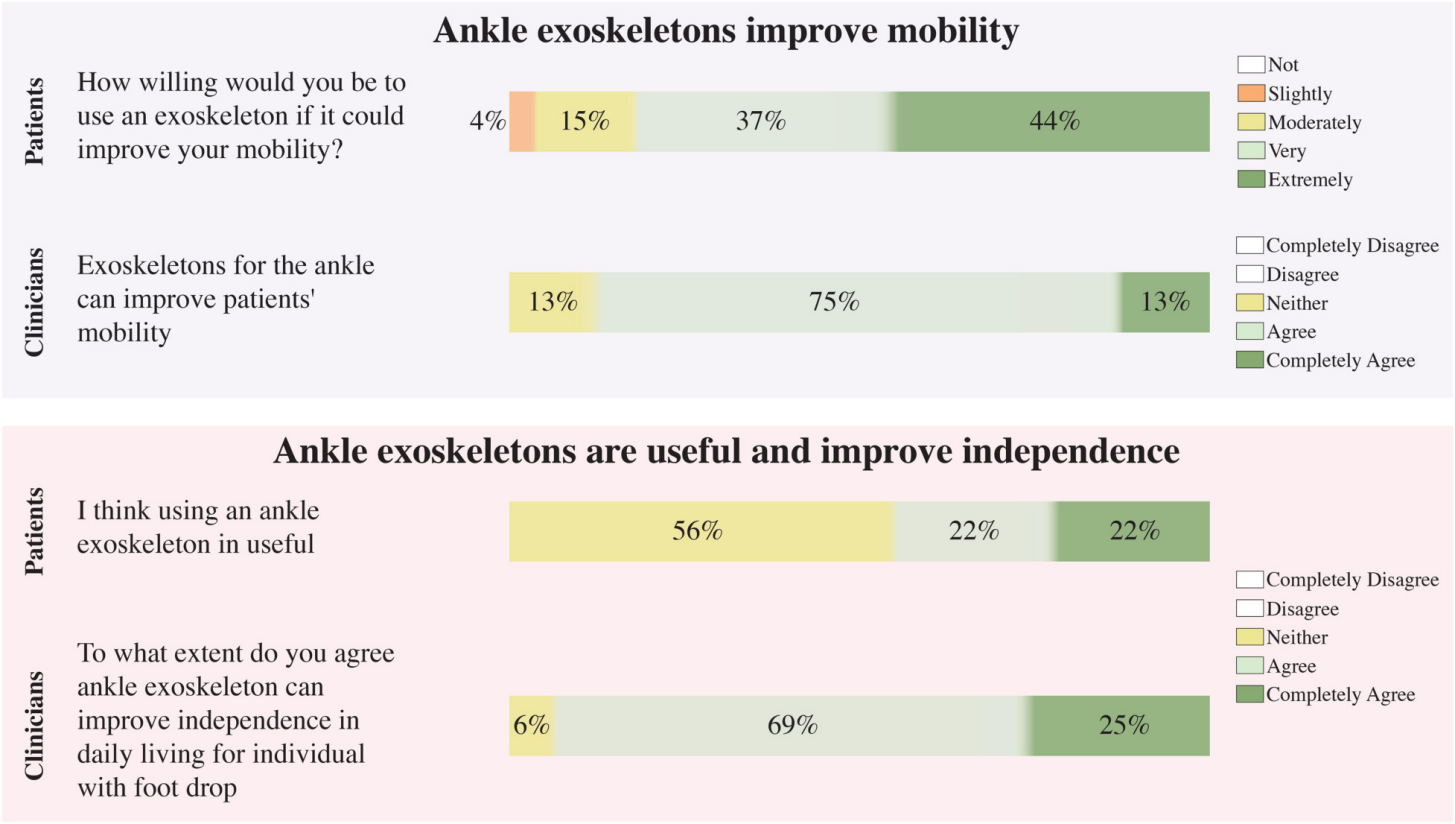
Patient and clinician perceptions of mobility and independence improvements associated with ankle exoskeleton use. The left side presents the questions used to assess each group’s knowledge. White boxes indicate unselected response options.

Regarding the attitudes of patients to improve mobility with an ankle exoskeleton, a significant majority (81%) expressed a strong willingness to use this device, with 44% being extremely willing and 37% very willing (see Figure 3 top). The remaining patients showed some degree of openness, with 15% somewhat willing and 4% slightly willing. Notably, no patients were unwilling to try the technology. For the clinicians, 87% of respondents agreed that the exoskeletons can improve mobility, with 12% strongly agreeing and 75% agreeing. The remaining 12% were neutral, with no disagreement reported (see Figure 3 lower).

Most foot drop patients (56%) held a neutral view regarding the usefulness of exoskeletons to assist them, while 44% perceived potential benefits (see Figure 3 bottom). On the other hand, clinicians reported a positive perception of 94% for independence improvement, with 25% strongly agreeing and 69% agreeing (see Figure 3 lower). Only 6% remained neutral, and again, no clinicians disagreed.

Normality assessment using the Shapiro–Wilk test indicated significant deviations from normality for both patient and clinician attitude scores (patients: W=0.81, p<0.001; clinicians: W=0.70, p<0.001). In this context, a Mann–Whitney *U* test was conducted to compare these attitude-related measures between groups concerning the statement that ankle exoskeletons improve mobility. Patients reported a median = 3, IQR = 3–4, indicating generally positive attitudes toward using an exoskeleton if it could enhance their mobility. Clinicians reported a similar level of agreement, with a median = 3, IQR = 3–3, reflecting consistent endorsement of the potential benefits of exoskeletons for patient mobility. The difference between groups was not statistically significant (U=264, p=0.18, r=0.20), suggesting a shared positive attitude across both groups.

**Figure 4:**
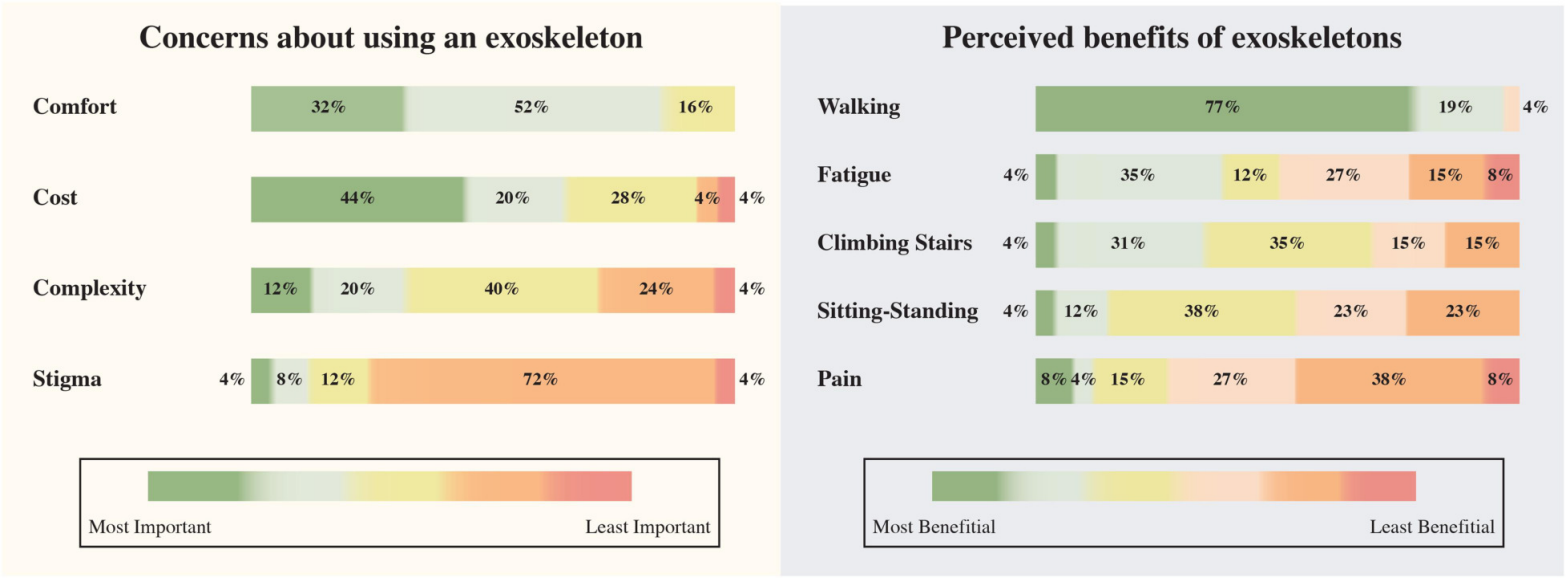
Patients’ concerns (left) and perceived benefits (right) regarding the use of ankle exoskeletons.

Patients identified comfort as the most significant concern (82%) regarding patient use of ankle exoskeletons, as Figure 4(left) shows. Cost followed as the second most relevant factor, cited by 64% of respondents. In contrast, complexity and stigma were considered less critical, with 32% and 12%, respectively. Figure 4(right) illustrates patients’ ranking of potential exoskeleton benefits for foot drop. Walking improvement is perceived as the most beneficial aspect, with 96% of patients ranking it highest. Fatigue and climbing stairs activity follow as the second and third most important benefits, with 39% and 35%. Sitting-standing transitions and pain reduction are less crucial for the patients.

**Figure 5:**
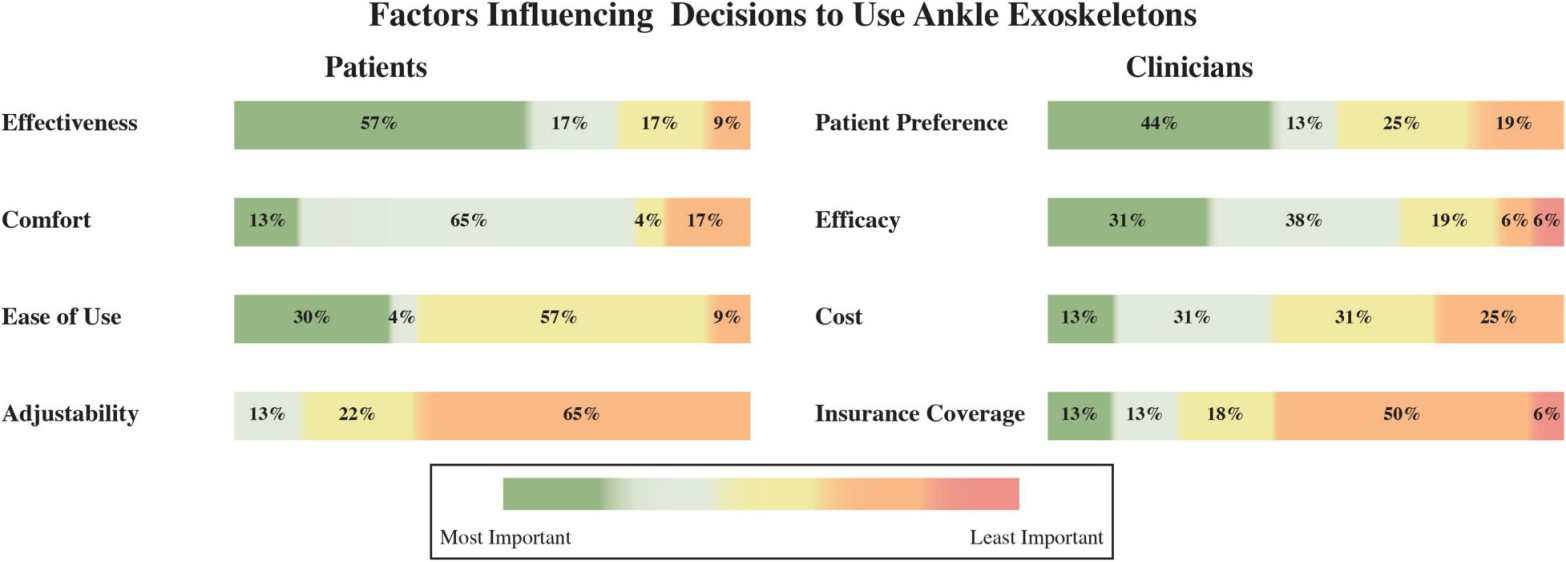
Factors influencing patients’ (left) and clinicians’ (right) decisions to use ankle exoskeletons in rehabilitation and daily activities.

Effectiveness emerges as the primary preference (see Figure 5 left), with 74% of patients ranking it in the top two tiers of importance (57% most important and 17% second most important). Comfort follows as the second most important preference (78% in total), with only 13% of participants considering it as most important. Preferences regarding Ease of Use were more evenly distributed, with 34% of respondents considering it an important aspect, 57% remaining neutral, and 9% rating it as the least important. Adjustability emerged as the least prioritised factor, with only 13% of patients identifying it as an important characteristic. Figure 5(right) illustrates clinicians’ practices regarding various factors influencing their decision-making. Clinicians reported patient preference and efficacy as the most significant factors (i.e., 44% and 31%, respectively). Cost considerations show a mixed impact, with 13% rating it highly important and 31% as moderately important. Insurance Coverage appears to be the least influential factor for the clinicians, with 50% rating it as relatively unimportant.

#### 4.1.2 Post-simulation

**Figure 6:**
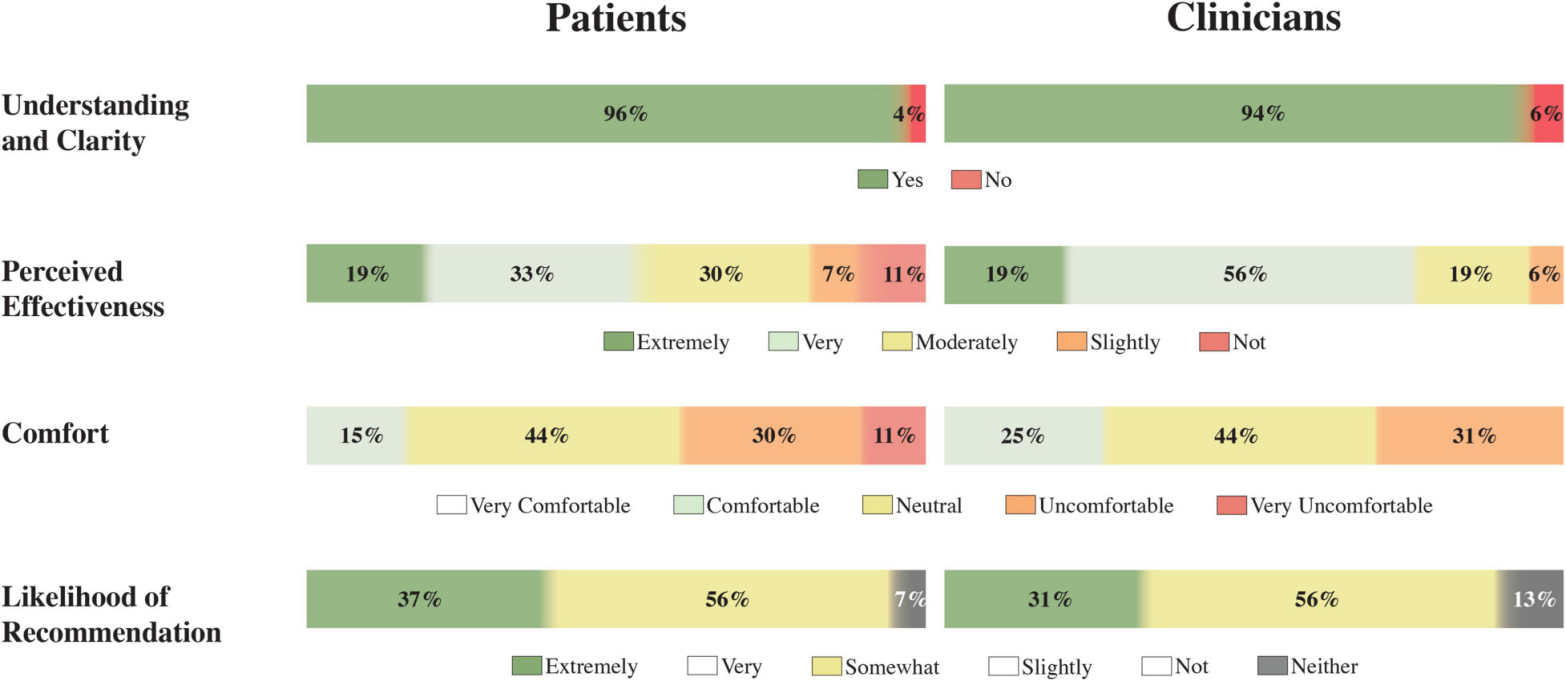
Perceptions of patients (left) and clinicians (right) regarding ankle exoskeletons’ use following the video simulation.

Regarding the video reactions, patients’ responses after this stage demonstrated a clear understanding of the exoskeletons’ function (96%, 26 out of 27 patients), as shown in Figure 6. Regarding perceived effectiveness for mobility, 52% of patients found it very or extremely effective, while 30% found it moderately effective. Similarly, 94% of clinicians (15 out of 16) reported a clear understanding of the exoskeleton technology. This finding is consistent with the positive perceptions of ankle exoskeletons’ effectiveness in improving mobility reported in the pre-simulation survey. However, these results are presented as descriptive observations across stages, as the measures assess different constructs and do not represent within-subject change. Comfort was the ankle exoskeletons’ lowest-rated aspect shown in the video session, with 41% of patients and 31% of clinicians reporting that the devices appeared uncomfortable or very uncomfortable. Moreover, both groups agreed somewhat with the recommendation to use this technology in daily living and rehabilitation activities.

In summary, patients rated the effectiveness of ankle exoskeletons as moderate on average, while clinicians rated it as very effective. Both groups expressed a neutral perception regarding comfort. However, when asked about their likelihood to recommend the technology, clinicians and patients rated it as likely. Regarding the most beneficial ADLs, both groups identified walking as the top choice. As the second most beneficial activity, patients selected stair climbing, while clinicians highlighted outdoor activities.

### 4.2 Thematic analysis

The qualitative component of this study explored patient and clinician perceptions through open-ended survey responses collected before and after the video simulation. A thematic analysis was conducted to identify common concerns, expectations, and suggestions related to ankle exoskeletons. Tables 2, 3 present a summary of the key themes, number of mentions, and illustrative comments from each group.

**Table 2:** Thematic analysis of patient responses on ankle exoskeleton use.

Timepoint	Thematic area	Theme	Mentions	Representative comment
Pre-video simulation	Fall prevention	Stability	3	–
		Safety/Skin protection	2	–
	Ease of use	Donning/doffing	5	–
		Wardrobe compatibility	4	“Multiple shoe connections… would lose patience.”
		Simplicity	3	–
		Extended wear comfort	2	–
	Decision	Cost/insurance	6	“Cost assurance… many braces… never work.”
		Proven effectiveness	4	–
		Quality of life impact	3	“If I could drive… I’m 100% wanting one…”
		Aesthetic concerns	3	–
Post-video simulation	Initial reaction	Size/complexity	6	“Way too big, too bulky…”
		Practicality concerns	5	–
		Hope for mobility	4	–
		Scepticism	2	–
	Design	Bulkiness	8	–
		Functionality appreciation	2	–
	Video concerns	Independent use	4	“I live alone and worry I won’t be able to get it on.”
		Learning curve	2	“Training time required.”
		Social visibility	2	–
	Improvements	Size/aesthetics/weight	11	“Make it smaller and lighter. Use carbon fibre.”
		Clothing/footwear fit	3	–

**Table 3:** Thematic analysis of clinician responses on ankle exoskeleton use.

Timepoint	Thematic area	Theme	Mentions	Representative comment
Pre-video simulation	Rehab concerns	Cost/accessibility	5	“Cost of acquisition and potential complications.”
		Learning curve	3	“Support of a technical person needed.”
		Skin/comfort issues	2	–
	Patient suitability	Condition/capabilities	4	“Assess medical history, severity, and needs.”
		Long-term goals	2	“Follow up on rehab outcomes.”
		Comfort/preference	2	–
Post-video simulation	Initial reaction	Excitement	6	“Overwhelmingly positive, interested to learn more.”
		Complexity/practicality	3	“Concerned about feasibility.”
		Curiosity/learning	2	“Excited to explore new health tech.”
		Benefits	2	“Pretty remarkable.”
	Design	Bulkiness	5	“Looks heavy and uncomfortable.”
		Complexity	2	“Genius idea but complex and error-prone.”
		Cosmetic appeal	2	“Needs aesthetic improvement.”
	Recommendation concerns	Affordability	3	“Unaffordable for lower-income patients.”
		Weight	2	“Concerned about equipment weight.”
	Improvements	Reduce size/weight	3	“Simplify and reduce bulk.”
		Better aesthetics	2	–

Table 2 outlines themes reported by patients. Their feedback highlighted priorities such as safety features (e.g., fall prevention and skin protection), usability factors (e.g., ease of donning/doffing and wardrobe compatibility), and conditions influencing their willingness to try the technology, including cost, effectiveness, and quality of life. Patients also commented on the perceived bulkiness of the devices and suggested design improvements, such as reducing size and enhancing comfort and aesthetic appeal.

Table 3 presents clinicians’ thematic responses. Clinicians focused on practical and implementation-related concerns, such as cost, device complexity, and patient suitability. Their insights also reflected an interest in the potential benefits of the technology, tempered by caution about its bulkiness and the need for cosmetic and functional refinements. Many emphasised the importance of matching device use to patient condition and long-term rehabilitation goals.

### 4.3 System usability scale (SUS)

Patients gave the exoskeleton an even lower average SUS score of 43.6, suggesting substantial room for improvement. Patients found the devices complex and potentially cumbersome, but appreciated the well-integrated functions and the ease of learning how to use the device. On the other hand, clinicians rated the ankle exoskeleton with an average SUS score of 54.1, which falls below the average SUS score of 68, indicating room for improvement in its design and user experience. The main drawbacks identified were the need for external support during therapies and the necessity to learn more about the exoskeleton before recommending it. Positive aspects included the use and recommendation of exoskeletons in practice and the well-integrated functions of these devices.

A Shapiro-Wilk test was conducted to assess the normality of SUS scores for patients and clinicians. With *p*-values of 0.25 for patients and 0.74 for clinicians, both exceeding 0.05, it was found that the SUS scores for these groups follow a normal distribution. Given this normality, an independent *t*-test was conducted to compare the means of the SUS scores between patients and clinicians. The *t*-test yielded a statistically significant difference between groups (t=2.53, p=0.016, d=0.81), with a 95% confidence interval for the mean difference of [2.05, 18.78]. This result indicates a statistically significant difference (p<0.05) between the SUS scores of patients and clinicians, suggesting that these two groups perceive the usability of the exoskeleton differently.

## 5 Discussion

Unlike most previous research, which primarily relied on user perception following direct interaction with the device, this study examines the evolution of perception in response to an educational intervention, without physical contact with the technology. This approach allowed for an investigation into how information influences acceptance and provided insight into how awareness can modulate user expectations. By integrating a pre/post awareness survey, the study highlights perceptual shifts, an aspect rarely addressed in existing studies. The following sections explore these findings in detail, focusing on patients’ and clinicians’ perceptions, as well as key barriers to the adoption of ankle exoskeletons and potential future approaches.

### 5.1 Patients perceptions of ankle exoskeleton technology

Patients’ perceptions were characterised by a mix of high interest and limited initial awareness. The study revealed a significant knowledge gap among patients, with 70% unaware of exoskeletons for foot drop treatment and 81% reporting no understanding of the technology. This lack of awareness likely results from limited public exposure to exoskeleton technology and absence of patient education about emerging rehabilitation options. Despite this initial unfamiliarity, patients showed high levels of interest, with 81% expressing a strong willingness to use an exoskeleton. However, this finding should be interpreted with caution, as the sample consisted of a relatively small group of participants (n=27) recruited through voluntary online participation, which may have attracted individuals more open to or interested in new rehabilitation technologies.

After watching the simulation, patients recognised potential benefits, particularly for walking (63% ranked it as the most important benefit) and climbing stairs. These perceptions are consistent with studies showing that ankle exoskeleton assistance can enhance propulsion and improve walking efficiency (26–28). Such findings support the potential of these technologies to improve gait performance and facilitate functional mobility during daily tasks. The effectiveness of the video simulation in improving understanding, with 96% of patients reporting clear comprehension post-video, underscores the power of visual demonstrations in patient education. This finding can guide the development of educational materials for patients, emphasising visual aids to explain complex technologies.

Comfort emerged as a significant concern, with 84% of patients ranking it as a top priority and 41% finding the exoskeleton uncomfortable or very uncomfortable after watching the video simulation. This perception may be partly influenced by the specific devices presented during the video session, which provided only a limited representation of current ankle exoskeleton technologies. Nevertheless, these responses indicate that comfort is a key expectation among potential users. The low average SUS score of 43.6 from patients further indicates challenges related to perceived usability of the technology. In line with previous studies, user-centred design remains essential to address these issues and improve acceptance of assistive devices (21, 22, 29).

These findings can directly inform the design and implementation strategies of ankle exoskeletons. Prioritising design features that facilitate comfort and enhance overall user experience in future iterations may significantly enhance patient acceptance. Additionally, the discrepancy between patient interest and concerns about comfort suggests a need for extended trial periods to allow patients to assess the long-term usability of exoskeletons in their daily lives.

### 5.2 Clinician perspectives on ankle exoskeletons

Clinicians’ perceptions of ankle exoskeletons for rehabilitation and independent living were generally positive, with 80% agreeing they could improve mobility and 60% considering they could enhance independence. This positive attitude aligns with findings from the study on lower-limb exoskeletons, which found that 60% of clinicians were “moderately” to “extremely” likely to use a lower-limb exoskeleton in a rehabilitation clinic setting in the next 5 years (29). It also aligns with literature suggesting that clinicians recognise the therapeutic benefits of such devices (17, 19, 30). Furthermore, the equal percentage of clinicians (94%) found ankle exoskeletons effective for mobility after watching the video simulation, suggesting that visual demonstration does not play a crucial role in shaping clinicians’ perceptions with knowledge on these devices.

Despite these positive perceptions, a significant gap exists between attitude and practical application. The fact that 56% of clinicians reported never having used ankle exoskeletons, with 37% rarely recommending them, points to barriers in implementation. These barriers likely include limited access to the technology in clinical settings, concerns about cost-effectiveness, comfort, and the need for specialised training. While several ankle exoskeleton systems have been developed and a small number are commercially available, their integration into standard rehabilitation settings remains limited, and they may not yet be practical or suitable for all clinical environments. The relatively low average SUS score of 54.1 from clinicians further indicates that usability issues contribute to the hesitation in adopting this technology.

Interestingly, a recent study on lower-limb exoskeletons provides additional insight into clinicians’ priorities for exoskeleton features. Clinicians ranked reducing falls, trips, or stumbles and enhancing balance on uneven surfaces as the most important features, followed by not causing muscle atrophy, increasing the user’s physical activity (29). This work aligns with our findings and emphasises the multifaceted benefits clinicians expect from ankle exoskeletons.

These findings could inform strategies to increase ankle exoskeleton adoption in clinical practice. Providing more hands-on training opportunities for clinicians could bridge the gap between theoretical knowledge and practical application. Additionally, addressing the usability concerns highlighted by the SUS score and focusing on the prioritised features identified in this study could make ankle exoskeletons more appealing for clinical use.

### 5.3 Barriers and future directions for ankle exoskeleton adoption

The recognition of cost, size, and weight as key barriers to adoption by patients and clinicians highlights the need for more streamlined and user-friendly exoskeleton designs. This is consistent with findings from Hohl et al. (31), Valleé (32), and Reicherzer et al. (20), which also highlight economic challenges in adopting advanced rehabilitation technologies. In addition, aesthetics emerged as a relevant aspect among some patients and clinicians, who expressed unease about the visual bulk and overall appearance of the device after watching the video simulation. This finding aligns with the outcomes reported in Orlando et al. (33), where a review of lower-limb orthotic devices highlights that, particularly for ankle–foot orthoses, aesthetics, appearance, and clothing integration are commonly reported concerns among users.

Comfort and potential skin issues emerged as additional concerns in this study, particularly in the context of long-term use. Moreover, the integration of the device with users’ daily wardrobe and activities posed practical challenges, highlighting the need for more versatile and adaptable designs. Notably, the persistence of comfort and usability concerns even after educational interventions suggests that theoretical understanding alone is insufficient to address all user apprehensions. These findings underscore the importance of user-centred design strategies, which could be valuable in further exploring and addressing the priorities identified in this study.

This study also demonstrated that educational interventions, particularly simulation videos, had a significant positive impact on patients’ and clinicians’ knowledge and attitudes. In summary, after watching the video session, patients and clinicians reported a clear understanding of the technology (i.e., 96% and 94%, respectively). This trend was also found in the studies by Shore et al. (18) and McDonald et al. (6), who emphasised the importance of informative exposure in reducing initial apprehension. This way, the improved understanding could potentially lead to changes in future practices, with clinicians feeling more confident in recommending exoskeletons and patients being more willing to adopt them in their rehabilitation routines.

## 6 Limitations

The sample size, consisting of 27 patients and 16 clinicians, while sufficient for our mixed-methods approach, may limit the generalisability of our findings to the broader population of patients and clinicians dealing with foot drop. This relatively small sample size also restricted our ability to perform more complex statistical analyses or subgroup comparisons. While similar sample sizes have been reported in previous studies focused on ankle exoskeletons, this does not mitigate the limitation, and the findings should therefore be interpreted with caution.

This study, based on online surveys and video simulations rather than hands-on experience with exoskeletons, may have limited the depth of insights into real-world challenges and benefits. Participants’ perceptions were based on brief video demonstrations that varied in technological maturity and production quality, included both commercially available systems and research-stage prototypes, and in some cases represented devices targeting different levels of impairment (e.g., bilateral lower-limb assistance), which may not fully capture the nuances of long-term exoskeleton use in daily life. The cross-sectional nature of the study prevents us from observing how perceptions might change over time with increased familiarity and use of the technology. Additionally, while our mixed-methods approach, combining quantitative survey data with qualitative thematic analysis, provided rich insights, it also introduced challenges in data integration and interpretation.

An additional limitation relates to the construction of the knowledge composite score. Although internal consistency was acceptable, further analysis indicated that the items did not reflect a strong unidimensional construct. Therefore, the composite score should be interpreted as an exploratory aggregation of related aspects rather than a single underlying variable.

The use of the System Usability Scale (SUS) for evaluating the perceived usability of the exoskeleton, while validated, may not capture all aspects of user experience specific to rehabilitation devices. Furthermore, the statistical comparison between clinician and patient SUS scores, while revealing a significant difference, should be interpreted cautiously due to the small and unequal sample sizes.

The demographic profile of the patient participants represents another limitation of this study. The majority of participants with foot drop were female (93%), with only a small proportion of male respondents (7%). Additionally, most participants were over the age of 50. This imbalance in gender representation and age distribution may have influenced the perceptions and responses reported in the study and may not fully reflect the diversity of the broader population of individuals experiencing foot drop.

Another limitation is that the study was conducted entirely within the United Kingdom, which may limit the generalisability of the findings to other geographic or socio-economic contexts—particularly in low- and middle-income countries, where healthcare infrastructure, access to assistive technology, and rehabilitation priorities may differ significantly.

The analyses performed in this paper did not account for potential covariates such as age, gender, or professional experience, which may influence perceptions of exoskeleton technology. Given the relatively small sample size and exploratory nature of the study, multivariate analyses were not performed.

Finally, because recruitment and participation were conducted entirely online, it was not possible to clinically verify participants’ medical conditions. As a result, the presence of stroke or clinically diagnosed foot drop among patient respondents could not be independently confirmed by a clinician, which represents a potential limitation in the reliability of the self-reported participant characteristics.

## 7 Conclusions

This study offers important insights into the perceptions, expectations, and usability considerations of ankle exoskeletons among two key stakeholder groups: patients with foot drop and rehabilitation clinicians. By employing a mixed-methods approach, the findings reveal both promising opportunities and persistent challenges in the clinical and everyday integration of this assistive technology.

A marked knowledge disparity was observed, with most patients unaware of exoskeletons before the study, in contrast to the moderate familiarity reported by clinicians. This gap highlights the need for targeted patient education and ongoing professional development to support informed engagement with emerging rehabilitation technologies. Despite differing baseline knowledge, both groups demonstrated generally positive attitudes toward the potential benefits of ankle exoskeletons, particularly in enhancing mobility and independence, an encouraging foundation for future implementation.

Nonetheless, several barriers to adoption were identified, including concerns around cost, accessibility, device complexity, comfort, and practical usability in daily living scenarios. These findings underscore the importance of a user-centred, multifaceted strategy that addresses technological refinement and systemic integration.

Importantly, the educational video intervention appeared effective in improving awareness and shaping perceptions, suggesting that similar tools may play a vital role in facilitating technology uptake. Qualitative feedback further emphasised the need for design improvements, namely reducing bulk and weight, enhancing comfort, and optimising aesthetics to promote social acceptability. Addressing these priorities will be critical to advancing ankle exoskeletons from experimental innovation to routine rehabilitation practice.

## Data Availability

The datasets presented in this study can be found in online repositories. The names of the repository/repositories and accession number(s) can be found in the article/Supplementary Material.
