# Supplementary material for: Patient and clinician perceptions, expectations, and usability of ankle exoskeletons for daily living: a mixed-methods survey study
Source: Front Digit Health. 2026 May 14;8:1745605. doi: 10.3389/fdgth.2026.1745605 (PMC13216185; doi:10.3389/fdgth.2026.1745605)
Supplement: Supplementary file 1 [file supplementaryfile1.docx]

# SUPPLEMENTARY MATERIAL

Examples of coding and theme development from patient and clinician open-ended responses:

| Group | Timepoint | Representative Response | Codes | Final Theme |
| --- | --- | --- | --- | --- |
| Patient | Pre-video | Multiple shoe connections if it takes too much time to fit and adjust, I would lose patience. A contraption that is stylish, lightweight and allows someone to wear a variety of fashionable shoes would make me happy. | Wardrobe compatibility; fitting difficulty; aesthetic preference; lightweight design | Wardrobe compatibility; Aesthetic concerns |
| Patient | Post-video | Way too big, too bulky. I only need help lifting my foot. Forthcoming exoskeletons, such as HyperShell, are MUCH more compact. | Bulkiness; excessive size; unnecessary complexity; preference for compact design | Size/complexity; Bulkiness; Size/aesthetics/weight |
| Clinician | Pre-video | The cost of acquisition and potential complications. | Cost barrier; implementation concern; risk of complications | Cost/accessibility |
| Clinician | Post-video | I think it’s a genius idea but it looks a handful with many seemingly complex parts that may leave it prone to malfunctions. | Positive appraisal; device complexity; practicality concern; risk of malfunction | Complexity; Complexity/practicality |

Associated Dataset:
The dataset supporting this analysis is available at:
https://doi.org/10.6084/m9.figshare.29110136
Dataset from a Mixed-Methods Survey on Clinician and Patient Perceptions of Ankle Exoskeletons for Daily Living
